# Transcriptional changes related to metabolic and iron acquisition strategies of avian pathogenic *Escherichia coli* are associated with embryonic survival during yolk sac infection

**DOI:** 10.3389/fcimb.2026.1797806

**Published:** 2026-07-15

**Authors:** Jingyao Wang, Yadav Sharma Bajagai, Yanwen Shao, Ghulam Asghar Sajid, Runsheng Li, Surya Paudel

**Affiliations:** 1 Department of Infectious Diseases and Public Health, Jockey Club College of Veterinary Medicine and Life Sciences, City University of Hong Kong, Kowloon, Hong Kong SAR, China; 2 School of Health, Medical and Applied Sciences, Institute for Future Farming Systems, Central Queensland University, Rockhampton, QLD, Australia; 3 Jockey Club College of Veterinary Medicine and Life Sciences Research Centre for Applied One Health Research and Policy Advice, City University of Hong Kong, Kowloon, Hong Kong SAR, China

**Keywords:** APEC, Escherichia coli, host-pathogen interaction, RNA-seq, transcriptomics

## Abstract

**Introduction:**

Avian pathogenic *Escherichia coli* (APEC) causes significant economic losses in poultry production, yet the molecular mechanisms underlying divergent infection outcomes remain poorly defined. This study aimed to elucidate the *in vivo* transcriptional profiles in APEC from infected chicken embryos compared to *in vitro* grown culture.

**Methods:**

Chicken embryos were experimentally infected with an APEC isolate. Subsequently, RNA sequencing was performed on APEC isolated from the yolk of experimentally infected embryos that either died or survived the infection, alongside *in vitro* grown controls. Differential gene expressions, functional enrichment analyses, small RNA profiling were conducted to identify key transcriptional changes. Weighted gene co-expression network analysis (WGCNA) was applied to detect bacterial gene modules associated with host outcomes.

**Results and discussion:**

In embryos that died, APEC exhibited a transcriptionally inferred hypermetabolic program, characterized by upregulation of 443 genes involved in anaerobic respiration, nutrient scavenging, and purine and arginine biosynthesis, consistent with increased replication and virulence potential. In contrast, APEC recovered from surviving embryos displayed a transcriptionally inferred low-energy state under host immune pressure, characterized by suppression of central metabolism pathways and concurrent upregulation of genes associated with iron sequestration, toxin production, and cell envelope remodeling. WGCNA suggested the association of low-oxygen respiration and anaerobic metabolism, Fe–S cluster assembly, and purine and arginine biosynthesis genes with host lethality, while siderophore-mediated iron acquisition correlated with host survival. Arginine metabolism showed a positive association with pro-inflammatory host responses, while flagellar motility was associated with both pro- and anti-inflammatory signatures, suggesting a modulatory role of metabolic and motility pathways on host immunity. The analyses indicated that APEC survival and pathogenicity are more dependent on metabolic adaptation and iron competition than individual toxin genes. Additionally, 77 small regulatory RNAs were identified, suggesting potential regulatory roles in *in vivo* adaptation.

These findings demonstrate the transcriptional plasticity of APEC during infection, highlighting that low-oxygen respiration and anaerobic metabolism, iron acquisition, arginine and purine metabolism and flagellar motility may contribute to bacterial survival strategies. These findings are consistent with recent host transcriptomic evidence showing mitochondrial dysfunction, hyperinflammation, and metabolic collapse in the yolk sac of infected embryos, suggesting that coordinated host-pathogen metabolic interactions influenced infection outcomes.

## Introduction

Avian pathogenic *Escherichia coli* (APEC) is a Gram-negative bacterium that causes colibacillosis in poultry, leading to high mortality and production losses, and often necessitating antimicrobial treatment (Abdelhamid et al., 2024). Colibacillosis can be seen in either localized or systemic forms, manifested by pathological lesions such as cellulitis, peritonitis, omphalitis, salpingitis, and femoral head necrosis, depending on the type and age of birds. Of these manifestations, a yolk sac infection (omphalitis) stands out as a major contributor to substantial economic losses in young birds worldwide. Infection of embryos can also lead to reduced hatchability and embryo mortality, making the threat of *E. coli* infection a significant concern in hatcheries as well (Hananeh et al., 2021). In recent years, prevention and control of this disease have become a major challenge due to the lack of effective vaccination options and the growing emphasis on reducing antibiotic use (Paudel et al., 2024).

The APEC strains are genetically heterogeneous, even within a single bird, and harbor a wide array of virulence-associated genes, including those encoding adhesins, toxins, siderophores, and serum resistance factors, which collectively facilitate host colonization, iron acquisition, immune evasion, and survival under stress (Palmieri et al., 2023). However, despite extensive genomic studies, the pathogenesis of colibacillosis remains elusive due to the ongoing challenge of genetically distinguishing APEC from commensal *E. coli* strains (Dziva and Stevens, 2008; Palmieri et al., 2023). Consequently, the APEC pathotype is typically defined by the pathological conditions in the host from which the strains are isolated, which does not always align with their genotypic profiles. Furthermore, systemic isolates are not necessarily more pathogenic than intestinal isolates (Johnson et al., 2022), complicating the search for genetic hallmarks of virulence. These challenges highlight the need to move beyond *in vitro* pathogen characterization and instead investigate *in vivo* bacterial responses during host infection to better understand the biology of *E. coli* infection.

During infection, bacterial gene expression changes drastically as pathogens adapt to host-derived stressors, immune responses, and nutrient limitations (Fang et al., 2016). Additionally, small RNAs (sRNAs), often aided by the Hfq protein, play critical post-transcriptional roles in regulating virulence and stress responses (Dutta and Srivastava, 2018; Waters and Storz, 2009). RNA sequencing (RNA-seq) has enabled comprehensive profiling of coding genes and regulatory sRNAs during infection (Westermann et al., 2017).

Recent transcriptomic analysis of host responses during APEC infection in chicken embryos has identified the yolk sac as an important site for immune activation, metabolic imbalance, and mitochondrial dysfunction, especially in embryos that die from the infection (Wang et al., 2026). These findings suggest that the outcome of infection is strongly affected by the host’s metabolic resilience and the regulation of inflammation within the yolk sac environment. However, the bacterial transcriptional changes that contribute to these different host outcomes are still not well understood. Therefore, we conducted RNA sequencing of APEC recovered from yolk of infected chicken embryos that either died or survived, alongside *in vitro* cultures, to elucidate bacterial transcriptional changes associated with divergent host outcomes and to provide a complementary perspective to existing host transcriptomic evidence in the same infection model. By integrating differential gene expression, small RNA profiling and weighted gene co-expression network analysis (WGCNA), we aimed to elucidate the molecular adaptations that underpin APEC pathogenesis during embryonic infection and to identify bacterial pathways associated with host survival or lethality.

## Results and discussion

### Sequencing data quality assessment and global transcriptomics

**Figure 1:**
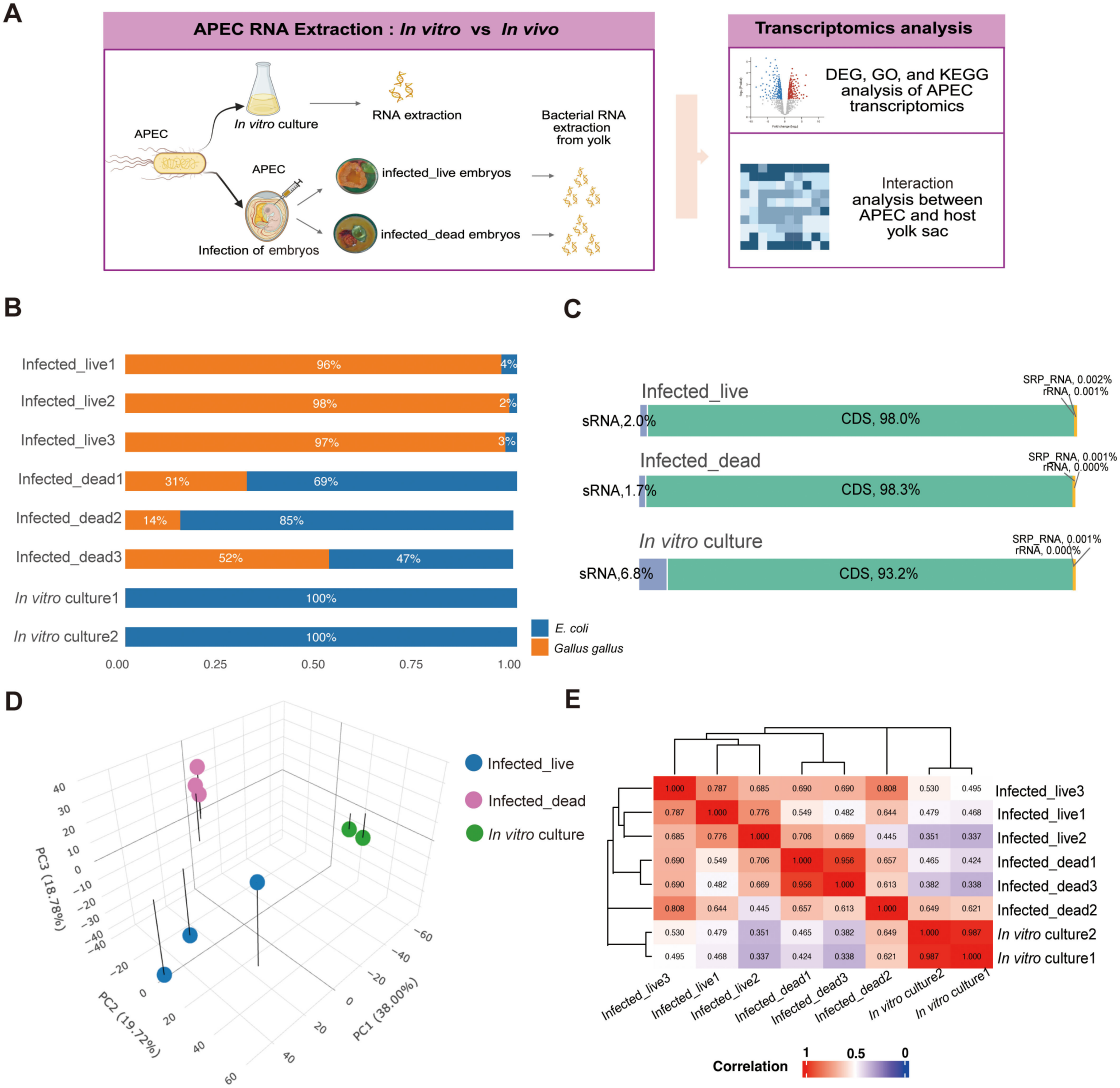
Overview of RNA sequencing analysis of APEC transcriptomes from *in vitro* and *in vivo* (infected-live and infected-dead) conditions. **(A)** Experimental design. **(B)** Summary of RNA-seq reads mapping statistics. **(C)** Distribution of RNA-seq reads assigned to different RNA classes **(D)** Principal component analysis (PCA) of normalized gene expression profiles from APEC from *in vitro* and *in vivo* conditions. **(E)** Heatmap of sample-to-sample correlations based on Pearson correlation of normalized APEC expression profiles.

The survival rate in the APEC-infected group was significantly lower than that in the negative control group (**Supplementary Figure S1**). By 48 h post-infection, cumulative mortality in the APEC-infected group reached 13/24 (54.2%). To investigate the transcriptional profiles in APEC during infection, we compared bacterial gene expression profiles in infected-live and infected-dead embryos relative to *in vitro* cultures (**Figure 1A**). Sequencing quality was high across all samples, with Q30 percentages ranging from 93.77% to 95.18%. Yolk samples yielded 80.85 to 95.67 million reads per sample (median, 86.78 million), whereas *in vitro* samples yielded 8.43 to 8.75 million reads (median, 8.59 million) (**Supplementary Table S1**). After quality filtering, the number of clean reads accounted for 95% to 97% of raw reads in each sample (**Supplementary Table S1**). In yolk samples from infected-live embryos, approximately 96.0% to 98.0% of reads were uniquely mapped to the *Gallus gallus* genome, whereas 2% to 4% were uniquely mapped to the bacterial genome in a single competitive alignment (**Figure 1B**; **Supplementary Table S1**). In contrast, yolk samples from infected-dead embryos showed a markedly different pattern, only 14% to 52% of reads originated from chicken, while 47% to 85% were mapped to the bacterial genome under the same alignment strategy (**Figure 1B**). While not a direct quantitative measure, this difference provides an indirect proxy for relative bacterial abundance within the yolk sac environment and suggests variation in bacterial burden between infection outcomes. However, this inference should be interpreted with caution, as RNA-seq read proportions are also influenced by RNA extraction efficiency, host RNA abundance, rRNA depletion efficiency, and bacterial transcriptional activity. Among bacterial transcripts, coding sequence (CDS) transcripts were predominant, comprising over 90% of the bacterial transcriptome. Other noncoding RNA classes, such as small RNA (sRNA), contributed approximately 2% of the bacterial transcripts from infected conditions, with a relatively higher proportion of sRNA observed in the *in vitro* culture (**Figure 1C**).

Principal component analysis (PCA) revealed separate clustering of APEC transcriptomic profiles in the *in vitro* culture, *in vivo* infected-live, and *in vivo* infected-dead groups, indicating substantial transcriptional differences between the experimental groups (**Figure 1D**). Furthermore, the Pearson correlation coefficients (r) of normalized gene expression between biological replicates ranged from 0.69 to 0.79 (infected-live), 0.61 to 0.96 (infected-dead), and 0.99 (*in vitro* culture) (**Figure 1E**). The inter-group correlation of normalized gene expression exhibited greater variability, with r values ranging from 0.45 to 0.81 between infected-live and infected-dead samples, 0.34 to 0.53 between infected-live and *in vitro* culture samples, and 0.34 to 0.65 between infected-dead and *in vitro* culture samples (**Figure 1E**). These results indicate that the *in vitro* culture environment differs substantially from the *in vivo* conditions, and samples from infected-dead and infected-live groups also display clear separation.

**Figure 2:**
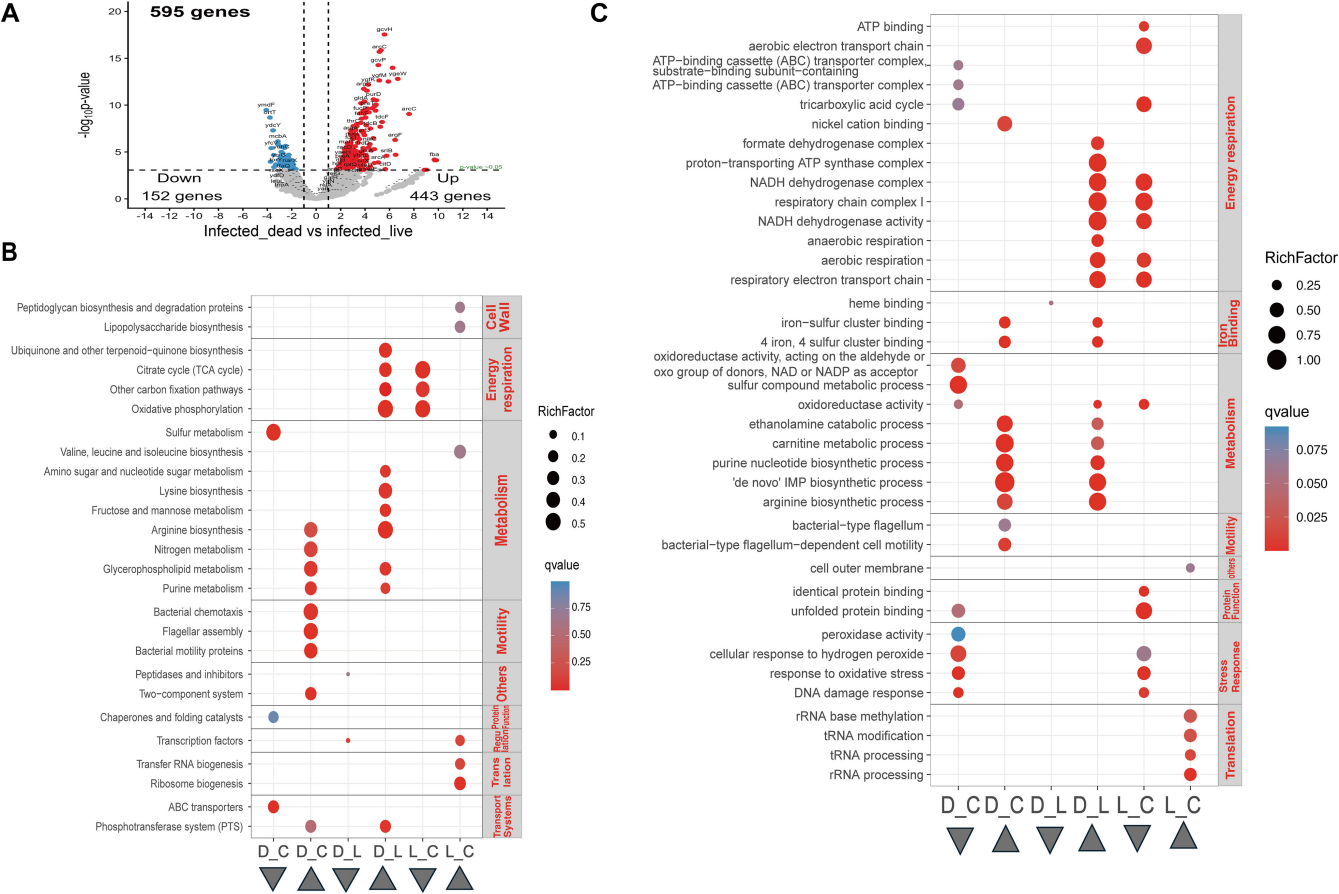
Transcriptomic profiling of APEC during infection in chicken embryos compared to *in vitro* culture. **(A)** Volcano plot illustrating the distribution of significantly up- and downregulated DEGs in APEC obtained from infected-dead versus infected-live embryos. **(B)** Kyoto Encyclopedia of Genes and Genomes (KEGG) pathway and **(C)** Gene Ontology (GO) enrichment analysis of differentially expressed genes (DEGs); D, infected-dead; L, infected-live; C, *in vitro* culture; ▲, upregulated; ▼, downregulated.

Compared to infected-live condition, 595 DEGs were differentially observed in infected-dead, with 443 upregulated genes and 152 downregulated genes (**Figure 2A**; **Supplementary Table S2**). These findings highlight a pronounced transcriptional shift in APEC in dead embryos, suggesting an adaptation to the altered host environment. KEGG and GO pathway enrichment analyses revealed that genes upregulated in APEC isolated from infected-dead embryos, compared to *in vitro* cultures, were enriched in pathways related to anaerobic bioenergetics, including iron-sulfur cluster binding and nickel cation binding, virulence-associated functions, such as flagellar assembly and bacterial motility, and key metabolic processes such as purine metabolism, arginine biosynthesis, and nutrient acquisition (e.g., glycerophospholipid, ethanolamine, and carnitine metabolism). These transcriptional changes support a metabolically active, invasive state characterized by enhanced anaerobic respiration (**Figures 2B, C**; **Supplementary Tables S3**, **S4**). While these transcriptional signatures are indicative of increased metabolic activity, direct functional validation (e.g., measurement of ATP levels, respiratory activity, or enzyme kinetics) was not performed. Recent complementary host transcriptomic analysis in the same embryo infection model revealed hypoxia, hyperinflammation, suppression of oxidative phosphorylation, and increased glycolysis in infected-dead embryos (Wang et al., 2026). These host-derived changes are consistent with a metabolically stressed and resource-limited environment, providing biological context that supports the observed bacterial transcriptional adaptations.

In contrast, genes involved in oxidative stress responses, DNA damage repair, oxidoreductase activity, and ABC transporter function were downregulated relative to *in vitro* cultures, suggesting reduced energetic investment in general stress responses and a reallocation of energy toward context-specific, virulence-promoting pathways (**Figures 2B, C**; **Supplementary Tables S3**, **S4**). These bacteria appeared to reprogram their metabolic pathways to cope with environmental challenges such as acidity, hypoxia, oxidative stress, and inflammatory mediators including lysozyme and cytokines within the yolk microenvironment.

In APEC isolated from infected-live embryos, compared with *in vitro* cultures, significantly upregulated genes were enriched in pathways related to rRNA and tRNA processing, transcriptional regulation, and the biosynthesis of lipopolysaccharide and peptidoglycan (**Figures 2B, C**; **Supplementary Tables S3**, **S4**). These findings suggest that, during colonization of live embryos, APEC prioritizes protein synthesis and fortifies its outer membrane structure, likely as an adaptive response to host-derived oxidative stress and antimicrobial peptides (AMPs). In contrast, downregulated genes were predominantly associated with energy-generating processes, including oxidative phosphorylation, the tricarboxylic acid (TCA) cycle, aerobic respiration, NADH dehydrogenase activity, ATP binding and identical protein binding (**Figures 2B, C**; **Supplementary Tables S3**, **S4**). This transcriptional response may highlight host immune effectors, such as ROS, lysozyme, AMPs, and acidic pH, drive the disruption of fundamental bacterial processes, including membrane integrity, energy metabolism and intracellular redox balance, thereby promoting bacterial clearance.

Comparative analysis of APEC from dead versus live embryos revealed that upregulated differentially expressed genes (DEGs) were enriched in pathways related to energy metabolism, including oxidative phosphorylation, aerobic and anaerobic respiration, NADH dehydrogenase activity, tricarboxylic acid (TCA) cycle, iron-sulfur cluster binding, formate dehydrogenase activity, and proton-translocating ATP synthesis. Additionally, key metabolic processes such as arginine biosynthesis and inosine monophosphate (IMP) biosynthesis, along with nutrient scavenging pathways involving glycerophospholipids, ethanolamine, fructose, mannose, and carnitine, were significantly upregulated (**Figures 2B, C**; **Supplementary Tables S3**, **S4**). These patterns suggest that bacteria in dead embryos are metabolically active and highly adaptive, optimizing energy production through both aerobic and anaerobic pathways, synthesizing nucleotides and amino acids for growth and virulence, and scavenging host-derived nutrients to support survival and colonization.

### The key virulence genes response to infection

#### Adhesion and motility

**Figure 3:**
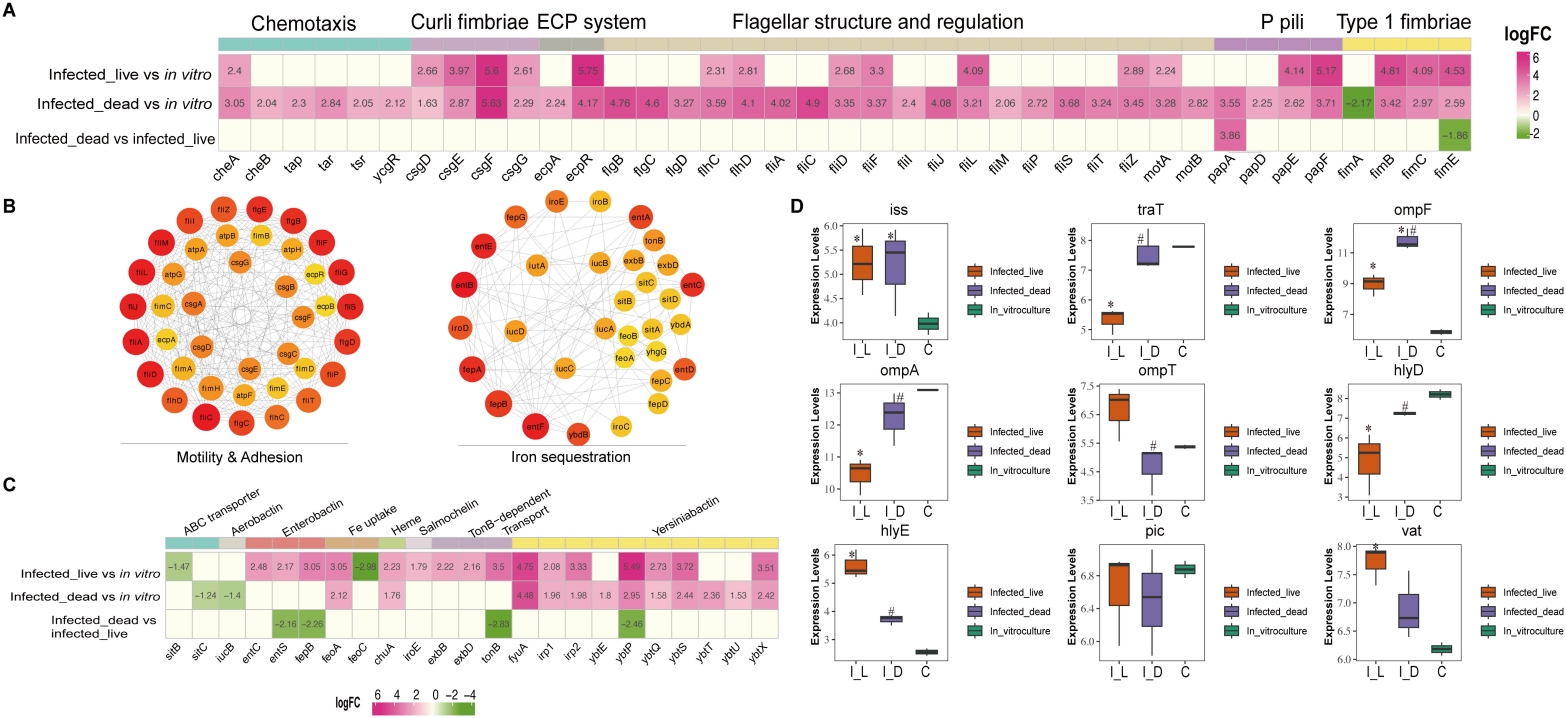
Comparative expression analysis of APEC virulence factors in infected-live, infected-dead embryos and *in vitro* culture. **(A)** Heatmap displaying the expression profiles of differentially expressed genes (DEGs) related to bacterial adhesion and motility. **(B)** Protein–protein interaction network based on genes associated with adhesion, motility, and iron sequestration. **(C)** Heatmap illustrating the transcriptional activity of genes involved in iron acquisition and sequestration. The numbers in the heatmaps indicate log2(fold-change) values, with blank cells representing genes with no statistically significant differential expression between groups. **(D)** Boxplots display the log-normalized expression levels of genes commonly associated with APEC virulence. Sample groups include I_D (infected-dead), I_L (infected-live), and C (*in vitro* culture). Asterisks (“*”) indicate significant differences between infected conditions (I_D or I_L) and *in vitro* culture **(C)**, while hash symbols (“#”) denote significant differences between infected-dead and infected-live conditions.

Although individual virulence-related traits such as motility and adhesion have been well described, this study provides further understanding by connecting bacterial transcriptional states with host survival outcomes. It also integrates metabolic pathways, iron acquisition, and host-pathogen interaction networks. In the infected embryos, *E. coli* exhibited broad upregulation of genes associated with adhesion and motility (**Figure 3A**). Similar patterns of P fimbriae (*pap*), type 1 fimbriae (*fim*) and curli fimbriae were seen in both infected-live and infected-dead, suggesting roles in host attachment and biofilm formation. Notably, a greater number of flagellar and chemotaxis-related genes were upregulated in infected-dead embryos (**Figure 3A**). These transcriptional patterns are consistent with enhanced motility and host interaction potential, although direct measurements of bacterial colonization were not performed in this study.

The network of these genes revealed the highly interconnected structure, with flagellar genes and curli genes forming dense interaction clusters (**Figure 3B**), indicating coordinated regulation of motility and adhesion-related pathways. Overall, these findings suggest that APEC can dynamically adjust its surface structures and motility systems in response to the host environment, potentially contributing to adaptation and persistence during infection. Flagellar gene expression is tightly regulated, with *FlhDC*, *FliA*, and *FlgM* as key transcriptional regulators (Sun et al., 2022). The APEC *fliG* mutant shows reduced adherence, invasion, and serum resistance in chicken embryos (Yin et al., 2021). The upregulation of curli fimbriae components *CsgA* exacerbated inflammation during ExPEC-induced sepsis (Bian et al., 2000; Tukel et al., 2005). Together, these findings highlight the complex interplay between flagellar and fimbria structures in modulating both host-pathogen interactions and immune responses.

#### Iron acquisition and metal homeostasis

In infected embryos, APEC exhibited significant upregulation of iron and nickel acquisition systems compared with *in vitro* conditions, indicating transcriptional adaptation to the *in vivo* host environment (**Figure 3C**; **Supplementary Table S2**). In particular, siderophore-mediated uptake systems, including yersiniabactin, salmochelin and aerobactin, along with the heme receptor *chuA*, were primarily expressed, especially in bacteria recovered from infected-live embryos (**Figure 3C**). This transcriptional response suggests that APEC encounters a highly iron-restricted environment imposed by the host. Bacterial siderophores not only function in iron scavenging to support microbial growth but also act as virulence factors and immune modulators. These transcriptional patterns are consistent with adaptation to conditions of limited metal availability within the host, although direct measurements of iron concentrations were not performed in this study.

Through iron chelation, siderophores can modulate host cellular pathways by stabilizing hypoxia-inducible factor 1-alpha (*HIF-1α*), upregulating pro-apoptotic genes and MAPK phosphatases, and inducing the secretion of pro-inflammatory cytokines such as interleukin-6 (IL-6), IL-8, and CCL20. Concurrently, they downregulate genes involved in DNA repair, mitosis, and cell cycle progression, thereby facilitating bacterial pathogenesis (Holden and Bachman, 2015). The knockout of iron-acquisition genes has been shown to attenuate the virulence of both APEC and UPEC in chickens (Caza et al., 2008; Gao et al., 2012). The yersiniabactin (Ybt) system showed the highest expression in our data (**Figure 3C**). In uropathogenic *E. coli*, disruption of key Ybt components, *FyuA*, *YbtP*, and *YbtQ*, significantly reduced virulence in murine UTI models (Brumbaugh et al., 2015; Koh et al., 2016). While traditionally viewed as a siderophore, Ybt also functions as a broad-spectrum metallophore, binding metals like copper (Robinson et al., 2018). Cu (II)-Ybt complexes protect against copper toxicity and exhibit SOD-like antioxidant activity, enhancing survival within phagocytes (Chaturvedi et al., 2014). These findings highlight Ybt’s dual role in metal acquisition and detoxification as a key virulence mechanism. The *sitABCD* operon, encoding an ABC transporter for iron and manganese, is highly expressed in both live and dead embryos during infection, though slightly lower than *in vitro* (**Supplementary Table S2**), possibly due to more efficient energy generation under aerobic and anaerobic conditions *in vitro* compared to the predominantly anaerobic host environment. Furthermore, relatively reduced siderophore expression in dead embryos suggests increased iron availability from host tissue damage in the hypoxic condition, including iron released from heme proteins like hemoglobin and myoglobin, thereby lessening the need for high-affinity iron acquisition (Ganz and Nemeth, 2015).

Notably, complementary host transcriptomic analysis in the same embryo infection model has demonstrated hypoxia, hyperinflammation, oxidative stress, and mitochondrial dysfunction in infected embryos (Wang et al, 2026). These conditions are known to influence metal homeostasis and are consistent with an environment that can limit bioavailable iron (Wang et al., 2010; Wessling-Resnick, 2010) thereby supporting the observed induction of bacterial iron acquisition systems.

In addition to iron acquisition, genes associated with hydrogen metabolism and anaerobic respiration, including the *NikABCDE* transporter, formate hydrogenlyase subunit *hycE*, Fe–S cluster-containing proteins HycB and HycF, and hydrogenase maturation genes (*hypABC*) were significantly upregulated in bacteria recovered from infected-dead embryos compared to *in vitro* culture (**Supplementary Table S2**). These enzymes support hydrogen metabolism under low-oxygen conditions, highlighting APEC’s adaptation to hypoxic host environments.

#### Other virulence factors

Beyond adhesion, motility and iron acquisition, we observed condition-dependent regulation of other virulence-associated genes. The serum survival protein gene (*iss*) and *ompF* were significantly upregulated in infected embryos compared to *in vitro* culture (**Figure 3D**). Both these genes are considered important during pathogenesis of colibacillosis (Nawaz et al., 2024). Genes involved in surface protection (*traT*), membrane stability and permeability (*ompF*, *ompA*), and the membrane fusion component of the Type I Secretion System (T1SS) responsible for exporting the HlyA cytotoxin (*hlyD)* were significantly upregulated in infected-dead embryos compared to infected-live embryos, whereas genes encoding the outer membrane protease (*ompT*) and the pore-forming cytotoxin (*hlyE*), which disrupt host cell membranes and induce cytotoxicity, were significantly downregulated in the same comparison (**Figure 3D**) (Ovi et al., 2023; Sarowska et al., 2019). The genes, *hlyE* and *vat*, which encode a vacuolating toxin, were significantly upregulated in APEC from infected-live compared to *in vitro* culture indicating its potential role in host cell injury and local tissue pathology (**Figure 3D**). Interestingly, although translation and energy metabolism were suppressed in bacteria isolated from live embryos (**Figures 2B, C**), the limited available energy appeared to be preferentially allocated to increased toxin secretion.

APEC upregulated *gsp* operon genes encoding the Type II secretion system (T2SS), particularly in infected-dead embryos (**Supplementary Table S2**), facilitating the translocation of a wide range of folded periplasmic proteins and effectors that contribute to host tissue degradation, colonization, nutrition acquisition, and immune evasion (Nivaskumar and Francetic, 2014).

### APEC metabolism adaptation to diverse host-derived microenvironments

#### Nutritional flexibility and amino acids utilization

**Figure 4:**
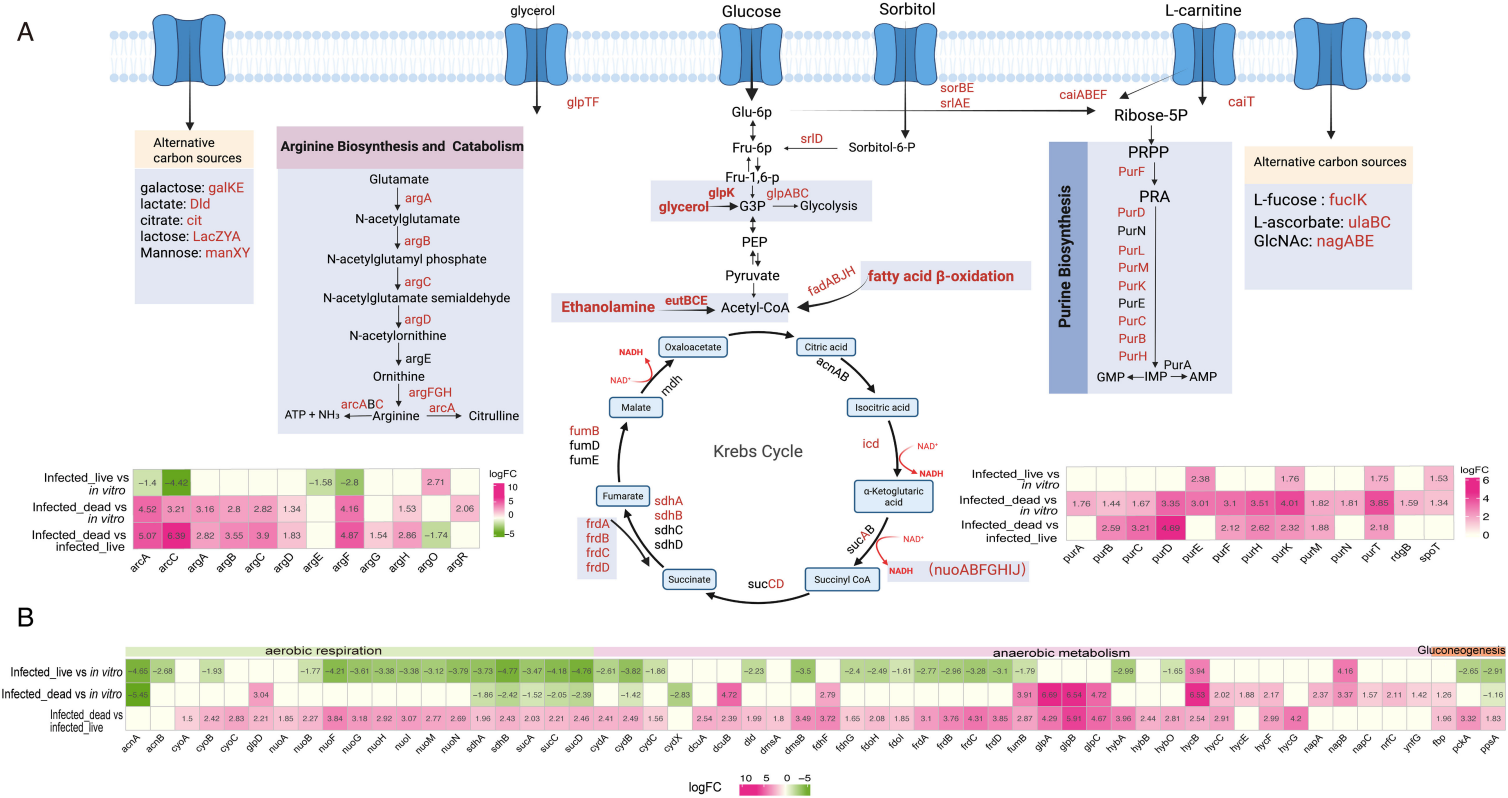
Metabolic alteration of APEC in different host microenvironments during infection. **(A)** Functional categorization of APEC genes significantly expressed (marked in red) in bacteria obtained from infected-dead compared to infected-live embryos. DEGs were manually curated and grouped into major functional categories, including alternative carbon source utilization, energy metabolism, arginine biosynthesis and catabolism, and purine biosynthesis. Important steps in different pathways are highlighted in gray boxes. **(B)** Heatmap representation of DEGs involved in energy generation metabolism under infected-live, infected-dead, and *in vitro* culture conditions.

Phosphotransferase System (PTS), a major bacterial sugar transport system, was activated in APEC from infected-dead embryos (**Figure 2B**). Transcripts encoding the glucose, sorbitol (*sorBE*, *srlABDE*), L-fucose (*fucIK*), L-ascorbate (*ulaBC*), GlcNAc (*nagABE*), galactose (*galKE*), L-carnitine (*caiABEF*), lactate (*Dld*), citrate (*cit* operon), lactose (*LacZA*) and fatty acid (*fadABJH*) uptake and metabolism systems were highly abundant in *E. coli* isolated from infected dead embryos compared to live embryos (**Supplementary Table S2**), reflecting an adaptive metabolic shift in *E. coli* toward the utilization of alternative carbon sources under glucose limitation, alongside a strategy for energy storage (**Figure 4A**).

Strikingly, genes involved in both the arginine biosynthesis pathway (*arg* operon) and the arginine deiminase (ADI) system (*arc* operon) (**Figure 4A**) were significantly upregulated in bacteria isolated from dead embryos compared to those from live embryos and *in vitro* conditions. Bacteria from the dead embryos could rapidly generate ATP from arginine via the ADI pathway, facilitating bacterial survival by neutralizing acidic host environments. The arginine succinyl transferase pathway encoded by the *ast* genes (*ast*ACDE) (**Supplementary Table S2**), which catabolizes arginine into glutamate and succinate were significantly downregulated during growth in the dead embryo than *in vitro* condition, indicating that arginine may not feed into this pathway. The elevated expression of arginine biosynthesis and catabolism genes in APEC is not unexpected, as similar results were shown in other pathogens, including UPEC and *Staphylococcus aureus* (Hibbing et al., 2020; Thänert et al., 2017). Notably, arginine auxotroph and mutants deficient in arginine biosynthetic genes exhibit significant growth defects both *in vitro* and *in vivo* (Chan and Lewis, 2022).

#### Carbohydrate and energy metabolism

In both live and dead embryo environments, TCA cycle genes and some terminal oxidases were downregulated relative to *in vitro* culture (**Figure 4B**), indicating that bacteria encounter hypoxic conditions. However, bacteria in dead embryos compensated by upregulating anaerobic respiratory pathways to sustain energy homeostasis and exhibited higher metabolic activity compared to infected-live embryos (**Figures 4A, B**). Specifically, transcription of key dehydrogenases, including NADH dehydrogenase I (*nuoA*–*nuoN*), formate dehydrogenase (*fdhF*), and hydrogenases (*hybA*, *hycBG*) oxidizing formate and hydrogen, was markedly upregulated, alongside elevated expression of terminal electron acceptors such as oxygen (*CydAB*), fumarate (*FrdABCD*), and DMSO reductase, supporting anaerobic energy generation (**Figure 4B**). Notably, *E. coli* from infected-dead embryos upregulated *glpABCQ* for glycerol utilization and *frdABCD* for fumarate respiration (**Figure 4A**, **Supplementary Table S2**). The significantly upregulated *eutBCE* indicates ethanolamine availability (**Figure 4A**), likely from host cell membrane phospholipid breakdown. Ethanolamine is a source of carbon and nitrogen for the bacteria.

Enrichment of the *RsxABC* complex suggests bacterial exposure to anaerobic oxidative and nitrosative stress relative to *in vitro* culture (**Supplementary Table S2**) (Imlay, 2013; Koo et al., 2003). The PPI network of energy production related genes revealed a densely connected core, particularly NADH dehydrogenase I (*nuo*) (**Supplementary Figure S2**).

The modular nature of *E. coli*’s respiratory chains allows them to be assembled in various configurations depending on the availability of terminal electron acceptors and the bacterium’s energy demands, reflecting the broader metabolic adaptability observed during APEC and UPEC infections (Subashchandrabose et al., 2014). This plasticity is important for maintaining energy homeostasis under respiratory stress and supports the use of alternative respiratory pathways that can modulate the proton motive force (PMF) to sustain ATP synthesis, solute transport, and motility (Shepherd et al., 2010; Unden and Bongaerts, 1997; Yang et al., 2023). Several of these complexes require iron–sulfur (Fe–S) clusters, which underscores the critical role of iron acquisition and ROS defense in APEC pathogenesis. Consistent with the importance of anaerobic respiratory capacity for *in vivo* fitness, mutational analyses of differentially expressed genes have demonstrated that formate dehydrogenase H and fumarate reductase are critical for the *in vivo* fitness of *Aggregatibacter actinomycetemcomitans* (Jorth et al., 2013). Markedly, central carbon metabolism and anaerobic respiratory chains are key determinants of ExPEC fitness in host environments, with evidence implicating cytochrome *bd* dependent respiration as a metabolic basis for intracellular bacterial pathogenesis (Beebout et al., 2022; Ma et al., 2020; Shepherd et al., 2016).

*Nucleic acid biosynthesis:* The coordinated upregulation of genes involved in *de novo* purine biosynthesis (the *pur* operon) was observed in bacteria isolated from infected-dead embryos (**Figure 4A**). These transcriptional changes reflect an increased cellular demand for purine nucleotides, likely driven by heightened requirements for purine biosynthesis and nucleotide turnover under nutrient-limited, oxidative, and proliferative conditions. Notably, studies using *in vivo* murine infection models have shown that deletion of the *purF*, which encodes glutamine phosphoribosylpyrophosphate amidotransferase, the rate-limiting enzyme in the *de novo* purine biosynthesis pathway, significantly impairs the growth and fitness of uropathogenic *E. coli* (Shaffer et al., 2017).

These adaptations reflect the nutritional composition of their host niches, bacterial competition for carbon and energy sources, and survival in the face of host defense mechanisms and stress, with the consistent findings from ExPEC studies in the urinary tract (Alteri and Mobley, 2012). In UPEC, transcriptomic and functional studies have reported rapid *in vivo* growth accompanied by strong iron acquisition, activation of nitrogen-limitation responses, adaptation to low-oxygen conditions, copper resistance, and broad metabolic reprogramming, collectively illustrating how ExPEC are adaptive during host–pathogen interactions (Torres-Puig et al., 2022). Our data align with the general ExPEC paradigm, and it has been demonstrated the importance of metabolic adaptation and iron acquisition for APEC during infection. In chickens, transcriptional profiling of APEC strain E058 showed broad *in vivo* metabolic activation relative to LB, including increased carbohydrate utilization and arginine metabolism, strong induction of iron acquisition and transport systems (e.g., *sit, chu, iuc*), and upregulation of genes supporting anaerobic respiration via alternative terminal electron acceptors; these trends closely mirror our view. Meanwhile, E058 showed downregulation of flagellar and fimbrial biosynthesis, differing from our findings (Gao et al., 2016). Similarly, a transcriptomic study of APEC O1 under serum exposure reported that the major transcriptional changes concentrated in iron acquisition, stress and defense responses, central metabolism, metal ion transport, and regulatory factors (Li et al., 2011). The deletions of genes including *dnaKJ*, *phoP*, *ybtA*, *flgE*, *tyrR*, *potF*, *yehD*, *ybjX, yjjQ*, *arcA* and *bfr* have been reported to attenuate APEC virulence during infection (de Paiva et al., 2016; Jiang et al., 2015; Li et al., 2008; Li et al., 2011). Targeting metabolic genes through genetic inactivation and iron acquisition pathways has therefore been proposed as a rational strategy for generating live attenuated vaccines against colibacillosis (Kwaga et al., 1994; Wang et al., 2023), potentially complementing and extending current control measures.

#### Reduction in respiratory function and bacterial membrane integrity

Bacteria from infected-live embryos exhibited significant dysregulation of genes encoding proteins involved in NADH dehydrogenase activity, respiratory function, protein binding, and ATP binding (**Figures 2B, C**), indicating a marked suppression of both aerobic and anaerobic respiratory metabolism, consistent with PMF reduction or collapse. These findings suggest that the bacteria undergo a shutdown of respiratory activity in response to unfavorable stress conditions encountered during host colonization and invasion (Passalacqua et al., 2016). Interestingly, bacterial metabolic reprogramming may represent an adaptive strategy to prioritize virulence over proliferation under such hostile conditions. The upregulation of virulence-associated genes (VAGs), including *iss*, *vat*, *ompF* toxins (**Figure 3D**), and iron acquisition systems (**Figure 3C**), is frequently accompanied by the downregulation of core metabolic pathways (**Figures 4A, B**), reflecting a trade-off between growth and pathogenicity.

Concurrently, in the infected-live embryo, the upregulation of genes involved in outer membrane repair (*pldA, pagP*) and peptidoglycan biosynthesis and remodeling (*mepS, mltC/mltF*) suggests the presence of outer membrane stress (**Supplementary Table S2**). A likely contributing factor is the host’s elevated production of antimicrobial peptides such as avian β-defensins and cathelicidins, or reactive oxygen species (ROS), and environmental stresses like acid shock, which may compromise membrane integrity, inhibit the activity of proton-pumping respiratory complexes and impair PMF maintenance.

### Altered abundance of conserved metabolic ncRNAs

In the *E. coli* EC-O119:H4 strain, we identified a total of 77 non-coding RNAs (ncRNAs) and the majority of which were trans-encoded sRNAs that predominantly interact with the RNA chaperone Hfq (**Supplementary Table S5**). This interaction is important in modulating the translation and stability of target mRNAs, often exerting negative regulatory effects under specific stress conditions (Gottesman, 2004). Compared to *in vitro* culture, 24 and 16 ncRNAs in infected-dead and infected-live embryos, respectively, were dysregulated and majority were downregulated (**Supplementary Figure S3**). Only 4 ncRNAs were differentially expressed when comparing bacterial transcript abundance between dead and live embryos (**Supplementary Figure S3**).

The carbon storage regulatory small RNAs, CsrB and CsrC, exhibited several-fold downregulation in *E. coli* isolated from infected embryos. These sRNAs antagonize CsrA by sequestering the RNA-binding protein, which modulates ribosome binding and mRNA stability (Babitzke and Romeo, 2007). The only upregulated sRNA in bacteria from dead embryos was 3′ETS-leuZ, a fragment from the 3′ external spacer of the glyW-cysT-leuZ tRNA precursor. Upregulation of 3′ETS-leuZ in dead embryos may therefore fine-tune regulatory sRNA networks to support the transcriptionally inferred hypermetabolic state observed in this condition (Lalaouna et al., 2015).

### Interactions between the APEC and host yolk sac transcriptomes

**Figure 5:**
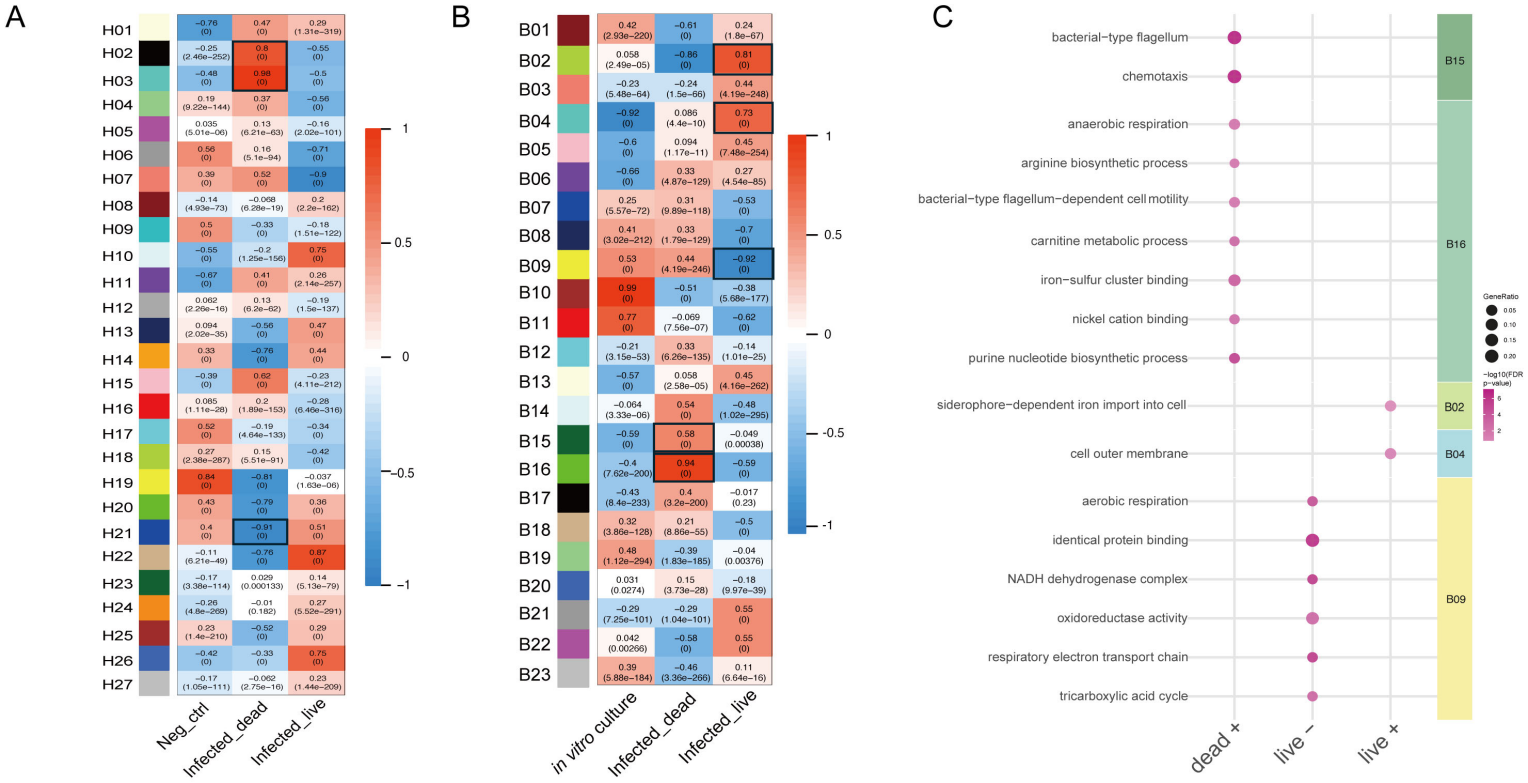
Correlation heatmaps of host and bacterial WGCNA modules. **(A)** Heatmap displaying Pearson correlation coefficients of host yolk sac gene co-expression modules (H01–H27) under different conditions: negative control (Neg_ctrl), infected-dead, and infected-live embryos. **(B)** Heatmap showing correlations of bacterial co-expression modules (B01–B23) derived from *in vitro* culture and *in vivo* environments (infected-dead, and infected-live). **(C)** Functional enrichment analysis of bacterial genes significantly correlated with “infected-dead” and “infected-live” embryo groups phenotype. Symbols indicate the direction of correlation, where “−” denotes a negative correlation and “+” indicates a positive correlation.

Weighted gene co-expression network analysis (WGCNA) of host and bacterial yolk sac transcriptomes revealed 27 host and 23 bacterial co-expression modules (**Figures 5A, B**; **Supplementary Table S6**). In the host, the “infected-dead” phenotype showed strong positive correlations with modules H03 (r = 0.98) and H02 (r = 0.80), enriched for inflammatory and extracellular matrix genes, and a strong negative correlation with H21 (r = –0.91), enriched for mitochondrial functions such as oxidoreductase activity and fatty acid β-oxidation (**Supplementary Table S7**). These patterns align with previous findings of heightened inflammation and disrupted mitochondrial and lipid metabolism in yolk sacs from dead embryos in the same model (Wang et al., 2026). However, these findings are derived from co-expression patterns and represent transcriptional associations rather than direct functional validation, therefore should be interpreted as indicative of coordinated regulatory responses rather than causal mechanisms.

In bacteria, modules B16 and B15, linked to purine biosynthesis, Fe–S cluster assembly, anaerobic respiration, flagellar motility, and chemotaxis, were most associated with the “infected-dead” group, suggesting enhanced virulence and adaptability (**Figures 5B, C**). Conversely, module B09, enriched for energy metabolism (TCA cycle, electron transport), was negatively correlated with the “infected-live” phenotype. Notably, *E. coli* from live embryos showed elevated expression of genes related to iron acquisition within module B02 (**Figures 5B, C**), possibly reflecting adaptation to host-mediated nutrient limitation.

As module B16 showed the strongest association with the bacterial “infected dead” phenotype, we focused on B16 and defined candidate hub genes using the following criteria: |MM| > 0.8, |GS| > 0.2, and weighted p< 0.05. Genes meeting these thresholds were intersected with DEGs identified from dead vs live or dead vs control comparisons, yielding 184 genes for downstream analyses. In parallel, we imported the node and edge tables for all genes in module B16 into Cytoscape, removed isolated nodes, and ranked genes using the MCC algorithm. Finally, intersecting the MCC top 180 list with the 184 DEG-overlapping candidates resulted in 103 prioritized genes (**Supplementary Table S8**). The prioritized gene set suggested the “infected dead” phenotype is associated with broad metabolic reprogramming and respiratory adaptation. The functional annotations of these genes were dominated by *de novo* purine biosynthesis (*pur* operon), glycerol utilization and transport (*glp* operon), and anaerobic respiration processes (e.g., *napAC*, *dmsAD*, *fdhF*, *hycAE*, *hybO,hypD*,*nikA-E*). In our analysis of the host network, two modules, H03 and H21, were identified as key features. Hub-gene inspection indicated two major functional signatures: immune and inflammatory regulation (e.g., *IL1B, IL6, TNF*, and NF-κB–related regulators) and fatty acid catabolism/energy production (e.g., *ACADL, HADH, ACAA*, and *ETFDH*) (**Supplementary Table S8**).

**Figure 6:**
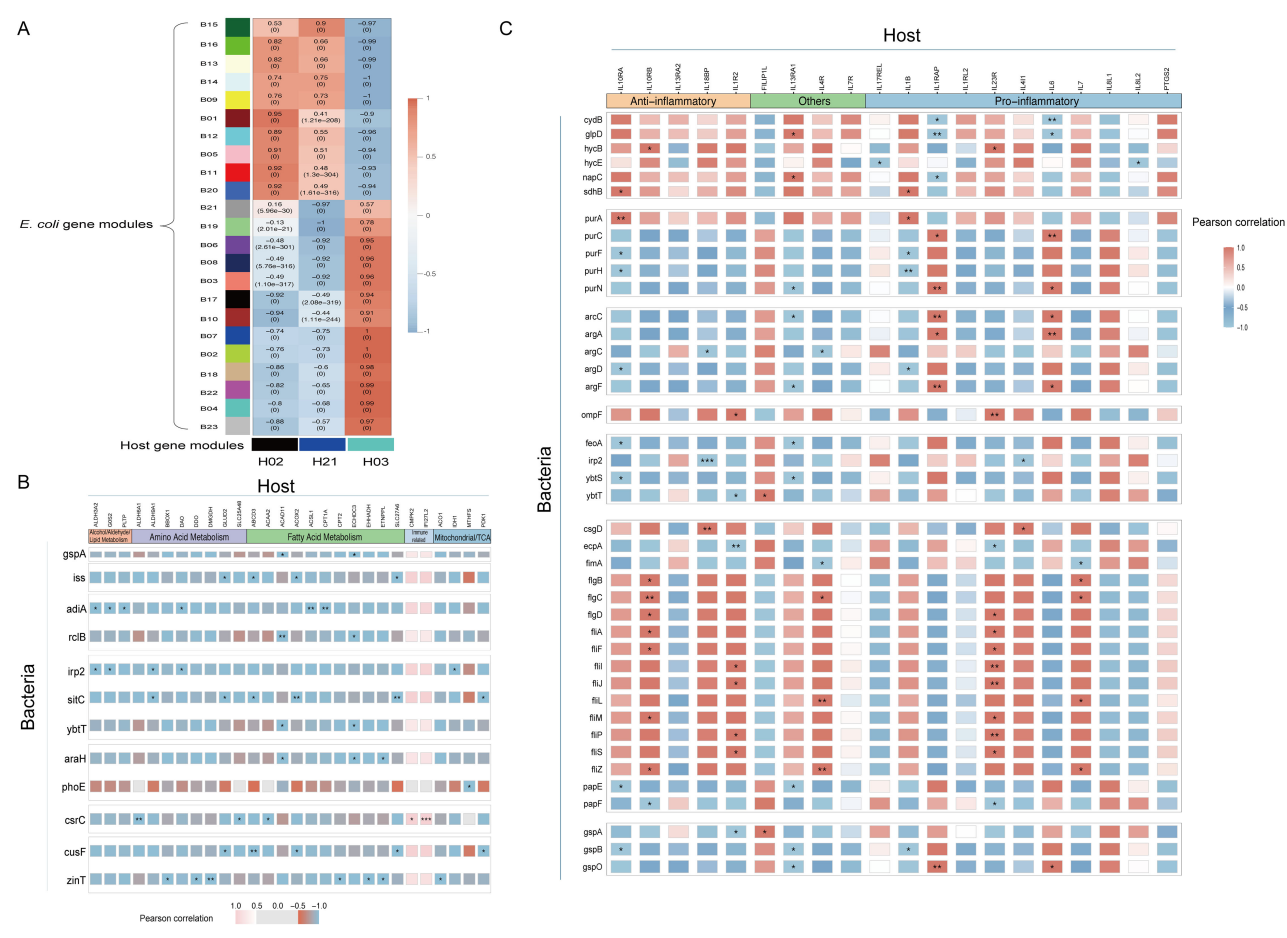
Transcriptomic correlation analysis between host and bacteria. **(A)** Pearson correlation heatmap illustrating associations between bacterial gene co-expression modules (B01–B23) and host yolk sac gene co-expression modules H02 (ECM module), H03 (inflammation module) and H21 (mitochondria module) derived from infected-dead embryos. **(B)** Heatmap illustrating Pearson correlation coefficients between DEGs from selected bacterial co-expression modules (rows) and host genes related with mitochondrial dysregulation (columns). **(C)** Pearson correlation heatmap between host inflammation related DEGs within H03 module (columns) and selected bacterial DEGs involved in virulence and key metabolic pathways (rows). Asterisks indicate statistical significance (*p ≤ 0.05; **p ≤ 0.01; ***p ≤ 0.001).

To further explore host-pathogen interactions in the “infected-dead” condition, we analyzed correlations between three host yolk sac transcriptome modules and bacterial WGCNA modules. The host H21 module, enriched for mitochondrial genes, showed strong negative correlations with bacterial modules such as B21, B19 (**Figure 6A**). Gene-level correlation analysis revealed that bacterial DEGs involved in metal ion transport (e.g., *cusF*, *zinT*), iron competition (*irp2*, *ybtT*), virulence (*iss*), acid resistance (*adiA*), secretion (*gspA*), and global regulation (*csrC*) were negatively associated with host mitochondrial genes (**Figure 6B**). Effector proteins such as EspF and Map secreted by enteropathogenic *E. coli* (EPEC) disrupt the cytoskeleton and trigger mitochondria-mediated cell death, a phenomenon also evident in yolk sac during APEC infection (Papatheodorou et al., 2006). The correlation analysis suggested the mitochondrial dysregulation may arise from multiple contributing factors, including known and potentially unidentified bacterial effectors. Specially, *zinT*, *irp2*, and *ybtT* may sequester essential cofactors like Fe²^+^ and Zn²^+^, while *adiA* and *gspA* may alter local pH or deliver toxic effectors. The cysteine protease espL1 which suppresses necroptosis and inflammation, was significantly upregulated in bacteria from infected live yolks compared to dead (Pearson et al., 2017). Given the central role of mitochondria in apoptosis, cellular energy production and innate immune, these interactions represented strategic mechanisms by which bacteria manipulated host cell physiology to promote their survival and persistence.

We conducted correlation analysis between bacterial genes from infected-dead embryos and host cytokine expression within the H03 module, which includes both pro- and anti-inflammatory genes (**Figure 6C**). Genes involved in respiratory metabolism (*cydB, glpD*) negatively correlated with pro-inflammatory cytokines like IL6, suggesting immune-mediated suppression of bacterial respiration. Iron acquisition genes (*irp2, feoA, ybtS*) showed negative correlations with anti-inflammatory cytokines, indicating potential antagonism between bacterial iron uptake and host immune regulation. Conversely, arginine biosynthesis genes (e.g., *arg* operon) were positively correlated with pro-inflammatory cytokines, implying a role in sustaining immune activation (**Figure 6C**). Flagellar genes correlated with both cytokine types, suggesting that motility structures may modulate immune responses and influence host–microbe interactions.

Despite providing valuable insights into host and bacterial transcriptional responses during embryonic APEC infection, this study has some limitations. Independent validation of selected differentially expressed genes by qRT-PCR or protein-level assays was not performed because limited RNA remained after sequencing, particularly for low-yield yolk samples. Nevertheless, the RNA-sequencing workflow and bioinformatics analyses employed in our study are robust, supported by previous research demonstrating strong correlations between RNA-seq results and qPCR validation, thereby providing confidence in the reliability and validity of our findings (Coenye, 2021). Although the findings are supported by coordinated transcriptional patterns, pathway-level enrichment, and stringent statistical thresholds, they should be interpreted as a transcriptional framework requiring further validation. In addition, bacterial load was inferred from sequencing data rather than direct CFU enumeration. Functional validation of metabolic reprogramming, and iron acquisition pathways was also beyond the scope of this study. Future studies using targeted qRT-PCR, protein-level assays, direct bacterial quantification, metabolomics, and functional validation will be important to confirm the mechanistic relevance of these findings.

## Conclusion

This study shows that APEC exhibits distinct and highly plastic transcriptional programs during embryonic infection that differ fundamentally from *in vitro* growth. In dead embryos, APEC activates a transcriptionally inferred hypermetabolic program marked by enhanced anaerobic respiration, broad nutrient scavenging, and upregulation of purine and arginine biosynthesis, potentially linked to rapid growth and virulence. The relationship between transcriptional activity and actual metabolic flux remains to be experimentally validated. In contrast, in live embryos APEC exhibits transcriptional signatures suggestive of reduced central metabolic activity and a stress-adapted, lower-energy state, while inducing virulence pathways such as iron sequestration, toxin production, and envelope remodeling. Consistent with this, previous studies show *E. coli* adapts its metabolism to host-like environments, favoring anaerobic growth and iron uptake in blood, but aerobic respiration and stress responses on solid media (Pettersen et al., 2016).

Growing evidence suggests that through metabolic adaptation to host-derived nutrients and signaling cues, bacterial pathogens engage in complex metabolic crosstalk that governs niche sensing, immune modulation, virulence expression, and ultimately infection outcomes (Olive and Sassetti, 2016; Passalacqua et al., 2016). In the context of APEC infection, our data indicated APEC survival and infection outcome were strongly associated with transcriptional signatures of metabolic adaptation and host–pathogen competition for iron than with single toxin-associated locus. When APEC exhibits transcriptional signatures consistent with increased activity, including iron acquisition, arginine and purine biosynthesis, energy generation, and carbon utilization, these patterns may reflect enhanced utilization of host-derived resources and may be associated with host signatures of mitochondrial perturbation and inflammation. In contrast, APEC recovered from surviving embryos exhibited transcriptional profiles consistent with host-imposed iron limitation, envelope stress responses, and reduced metabolic activity, suggesting a lower-energy, stress-adapted state (Bhagwat et al., 2025; Nüse et al., 2023; Shaffer et al., 2017). Overall, our findings support a model in which APEC infection outcomes are more strongly associated with transcriptional programs related to metabolic dominance and iron competition and identify bacterial energy metabolism and nutrient acquisition pathways as attractive promising directions for future mechanistic studies and intervention development.

## Materials and methods

### Bacterial strain and preparation of inoculum

The *E. coli* strain EC-O119:H4 was isolated from the liver of a broiler chicken exhibiting anorexia, dry shanks, pasty vents, hepatitis and omphalitis (Wang et al., 2025). The affected flock experienced a cumulative mortality rate of approximately 30% by 10 days of age. The isolate was cultured in Luria-Bertani (LB) broth at 37 °C for 12 hours under aerobic conditions and an aliquot was used for RNA extraction. For experimental infections, the bacterial culture was diluted in sterile phosphate-buffered saline (PBS) to a desired concentration, and bacterial counts were determined by colony-forming unit (CFU) counts in serial dilutions both before and after inoculation.

### Embryo inoculation

Specific-pathogen-free (SPF) fertilized chicken eggs were incubated under standard conditions (37 °C, 65% relative humidity). On day 12 of incubation, twenty-four eggs were inoculated with 100 μL of *E. coli* EC-O119:H4 suspension (approximately 5 × 10³ CFU/mL) via the allantoic sac. In parallel, sixteen eggs in the control group were inoculated with 100 μL of sterile PBS using the same route. Embryo viability was monitored every 8 hours by candling and nonviable embryos were discarded. Eggs were regarded viable when a clear, branching vascular network with visible embryonic movement was observed; non-viable eggs showed reduced vascularization (e.g., sparse or fragmented vessels) and no detectable movement.

At 48 hours post-infection, dead and live embryos from the infected group were sampled. Prior to sampling, the outer shell surface was disinfected with 70% alcohol before opening the eggs. Subsequently, yolk samples from infected-live and infected-dead were collected in sterile tubes. Kaplan–Meier survival curves were generated for the control and APEC-infected groups, and survival distributions were compared using the log-rank (Mantel–Cox) test.

### RNA extraction

Total RNA was extracted from *E. coli* grown in LB (*in vitro*) and from yolk samples of infected embryos (representing *in vivo* environment; dead and live conditions). Bacterial suspensions were lysed with QIAzol reagent (Qiagen) at a 4:1 ratio and homogenized using a Precellys^®^ homogenizer with 0.1 mm ceramic beads. Yolk RNA was isolated using the miRNeasy Mini Kit (Qiagen) following the manufacturer’s instructions. For transcriptomic analysis, three biological replicates from infected-live and infected-dead embryos, along with two *in vitro* samples, were used. RNA quality and concentration were assessed using the Agilent TapeStation system. The number of biological replicates was decided based on practical limitations of the embryo infection model and the amount of RNA obtained from yolk-derived bacterial samples. Although the *in vitro* group had a smaller number of replicates, high sequencing depth and good biological reproducibility were still achieved, as shown by principal component analysis and correlation metrics.

### RNA library preparation and sequencing

For the RNA samples from the infected embryos were subjected to ribosomal RNA (rRNA) depletion targeting both eukaryotic and prokaryotic rRNAs using the Illumina Ribo-Zero Gold rRNA Removal Kit (Epidemiology). Total RNA from bacteria cultured *in vitro* was subjected to ribosomal RNA depletion targeting only prokaryotic rRNAs to enrich for messenger RNA. Strand-specific cDNA libraries were constructed using the NEBNext^®^ Ultra II RNA Library Prep Kit for Illumina (New England Biolabs, USA), following the manufacturer’s guidelines. Paired-end sequencing (2 × 150 bp) was performed on the Illumina NovaSeq platform. All library preparation and sequencing procedures were conducted by Novogene Co., Ltd. (Beijing, China).

### Data processing and differential gene expression analysis

Quality of raw sequences was assessed with FastQC v0.11.8 (Andrews, 2010). Adapter sequences and low-quality reads (Q ≤ 20) were trimmed using Trimmomatic v0.39 (Bolger et al., 2014). The cleaned reads were mapped to both the *Gallus gallus* reference genome (GenBank accession: GCA_016699485.1) and the *E. coli* EC-O119:H4 genome (GenBank accession: CP162393-CP162398) using TopHat2 v2.1.1 (Kim et al., 2013). Differential gene expression (DEG) analysis was carried out using the edgeR package (Robinson et al., 2010), which is suitable and robust even for small sample sizes. Gene expression values were normalized using the trimmed mean of M-values (TMM) method. DEGs were defined based on a false discovery rate (FDR) ≤ 0.05 and an absolute log2 fold change ≥ 1.

### Functional enrichment analysis

To interpret the biological roles of DEGs, Gene Ontology (GO) and Kyoto Encyclopedia of Genes and Genomes (KEGG) pathway enrichment analyses were conducted using the clusterProfiler package (v4.5.0) in R (Yu, 2024). GO annotations were retrieved from the *E. coli* GO annotation release, and KEGG annotations were obtained directly from the KEGG database. Gene identifiers were converted using the Prokaryotic Genome Annotation Pipeline (PGAP) annotation. The compareCluster function (clusterProfiler, R) was used to identify enriched GO and KEGG terms using the enricher method with custom GO and KEGG annotation sets. GO enrichment was evaluated separately for the three ontology categories: Biological Process (BP), Molecular Function (MF), and Cellular Component (CC). Enrichment results were visualized as dot plots using the dotplot function in clusterProfiler. Statistical significance was assessed using the *p*-value <0.05, with multiple testing correction via the Benjamini-Hochberg method.

### Bacterial non-coding RNA (ncRNA) transcriptome analysis

Due to incomplete annotation of ncRNAs in the *E. coli* EC-O119:H4 genome, additional ncRNA features were annotated by aligning known *E. coli* K-12 MG1655 ncRNA sequences (GenBank: GCA_000005845.2) using BLASTn. A custom GFF3 file incorporating the extended ncRNA annotations was manually curated. Transcript quantification of bacterial ncRNAs was conducted using htseq-count with the updated GFF3 annotation.

### Weighted gene co-expression network analysis (WGCNA)

To investigate transcriptional coordination and its association with phenotypic traits, we constructed gene co-expression networks using the WGCNA package in R (Langfelder and Horvath, 2008), based on TMM-normalized gene expression matrices from chicken yolk sacs (GEO: GSE300118) and APEC transcriptomes. Soft-thresholding powers of 15 (APEC) and 11 (chicken) were chosen to meet scale-free topology criteria. Modules were identified using the blockwiseModules function (min module size = 30, mergeCutHeight = 0.25). Module eigengenes, representing the first principal component of each module, were correlated with experimental traits to identify relevant associations. A total of 23 modules were defined. Functional enrichment was performed using compareCluster (clusterProfiler), and hub genes were inferred based on high module membership (MM) and gene significance (GS). For key modules from infected-dead yolks, eigengenes were correlated with bacterial transcriptomes from the same condition, and Pearson correlation heatmaps were generated from normalized expression values.

### PPI network analysis

A protein–protein interaction (PPI) network was constructed for genes related to motility/adhesion, iron sequestration, arginine and purine metabolism, and energy/carbohydrate metabolism using STRING v12.0 (Szklarczyk et al., 2019). STRING settings were interactions filtered at high confidence (combined score ≥ 0.70), network type set to both functional and physical protein associations, and edges interpreted as interaction evidence. The resulting network was imported into Cytoscape v3.10.4 (Shannon et al., 2003) and hub proteins were identified using the Maximal Clique Centrality (MCC) algorithm via the CytoHubba plugin, chosen for its superior performance in detecting both high- and low-degree essential proteins (Chin et al., 2014). It provides 12 topological ranking algorithms to prioritize hub genes and network visualization showed that nodes with higher MCC scores were displayed in darker colors, whereas nodes with higher degree were shown as larger nodes, indicating a more central topological role in the PPI network.

## Data Availability

The RNA sequencing datasets generated in this study have been deposited at the National Center for Biotechnology Information Short Read Archive, under BioProject accession code PRJNA1378465. The corresponding SRA run accession numbers are SRR36461653 - SRR36461660.

## Supplementary material

Supplementary Figure S1 Survival of chicken embryos following APEC infection. Embryo survival was monitored at 8-h intervals by candling from 0 to 48 h post-infection. Kaplan–Meier survival comparison was analyzed between the negative control and APEC-infected groups (Control, n = 16; APEC, n = 24). Curves were compared using the log-rank (Mantel–Cox) test (p = 0.00053).

Supplementary Figure S2 Protein–protein interaction (PPI) networks of genes involved in selected metabolic pathways. The networks depict interactions among genes associated with arginine metabolism, purine metabolism, and energy and carbohydrate metabolism. Node size indicate degree centrality, with red representing highly connected proteins.

Supplementary Figure S3 Heatmap showing the differential expression of sRNAs in APEC from *in vivo* (infected-live and infected-dead) compared to *in vitro* culture. Log_2_ fold change (logFC) values that are statistically significant are shown.

Supplementary Table S1 Summary of *E. coli* RNA-seq results from yolk samples of infected embryos and *in vitro* cultures. Complete description of RNA-seq quality metrics and mapping statistics. Data include read counts, Q30 scores, clean reads and mapping ratios to *Gallus gallus* and *E. coli* genomes.

Supplementary Table S2 Differentially expressed genes (DEGs) identified by edgeR analysis in APEC transcriptomes from infected-dead embryos, infected-live embryos and *in vitro* cultures. Genes were filtered to retain those with expression counts >5 in at least two samples. Normalization was performed using the TMM method. DEGs were identified by exact tests with Benjamini–Hochberg correction (FDR < 0.05) and |log_2_ fold change| ≥ 1. Genes meeting these criteria are labeled “0sig” in the *isdeg* column; others are marked as “nosig”.

Supplementary Table S3 KEGG pathway enrichment analysis for APEC transcriptomes. The pathway ID, name, gene and background ratios, enrichment statistics (RichFactor, FoldEnrichment, z-score), and significance values (*p*-value, adjusted *p*-value, q-value), gene ID and counts were included.

Supplementary Table S4 Functional enrichment of Gene Ontology (GO) terms associated with differentially expressed genes (DEGs) in APEC. GO term ID, description, gene and background ratios, enrichment metrics (RichFactor, FoldEnrichment, z-score), and significance values (p-value, adjusted p-value, and q-value), gene ID and counts were included.

Supplementary Table S5 List of differentially expressed small RNAs (sRNAs) identified in APEC transcriptomes.

Supplementary Table S6 List of host yolk sac and bacterial genes assigned to WGCNA co-expression modules. **(A)** Genes from yolk sac samples assigned to WGCNA-derived co-expression modules. **(B)** Genes from APEC samples assigned to WGCNA-derived co-expression modules.

Supplementary Table S7 Gene Ontology and KEGG enrichment of host and bacterial WGCNA modules. Enriched GO terms **(A)** and KEGG **(B)** associated with host genes in yolk sac WGCNA modules. Enriched GO terms **(C)** and KEGG **(D)** associated with bacterial genes in APEC WGCNA modules.

Supplementary Table S8 Comprehensive gene expression (APEC and yolk sac) including module assignment and correlation with phenotypic traits derived from WGCNA analysis. Values in the table presents Pearson correlation coefficients between the expression of each gene and the eigengenes of WGCNA modules and phenotypic traits, along with the corresponding p-values indicating the statistical significance of each correlation. Hub genes within the module are highlighted to emphasize their central regulatory roles.
