# Supplementary figures and images for: Transcriptional changes related to metabolic and iron acquisition strategies of avian pathogenic Escherichia coli are associated with embryonic survival during yolk sac infection
Source: Front Cell Infect Microbiol. 2026 Jul 15;16:1797806. doi: 10.3389/fcimb.2026.1797806 (PMC13414258; doi:10.3389/fcimb.2026.1797806)

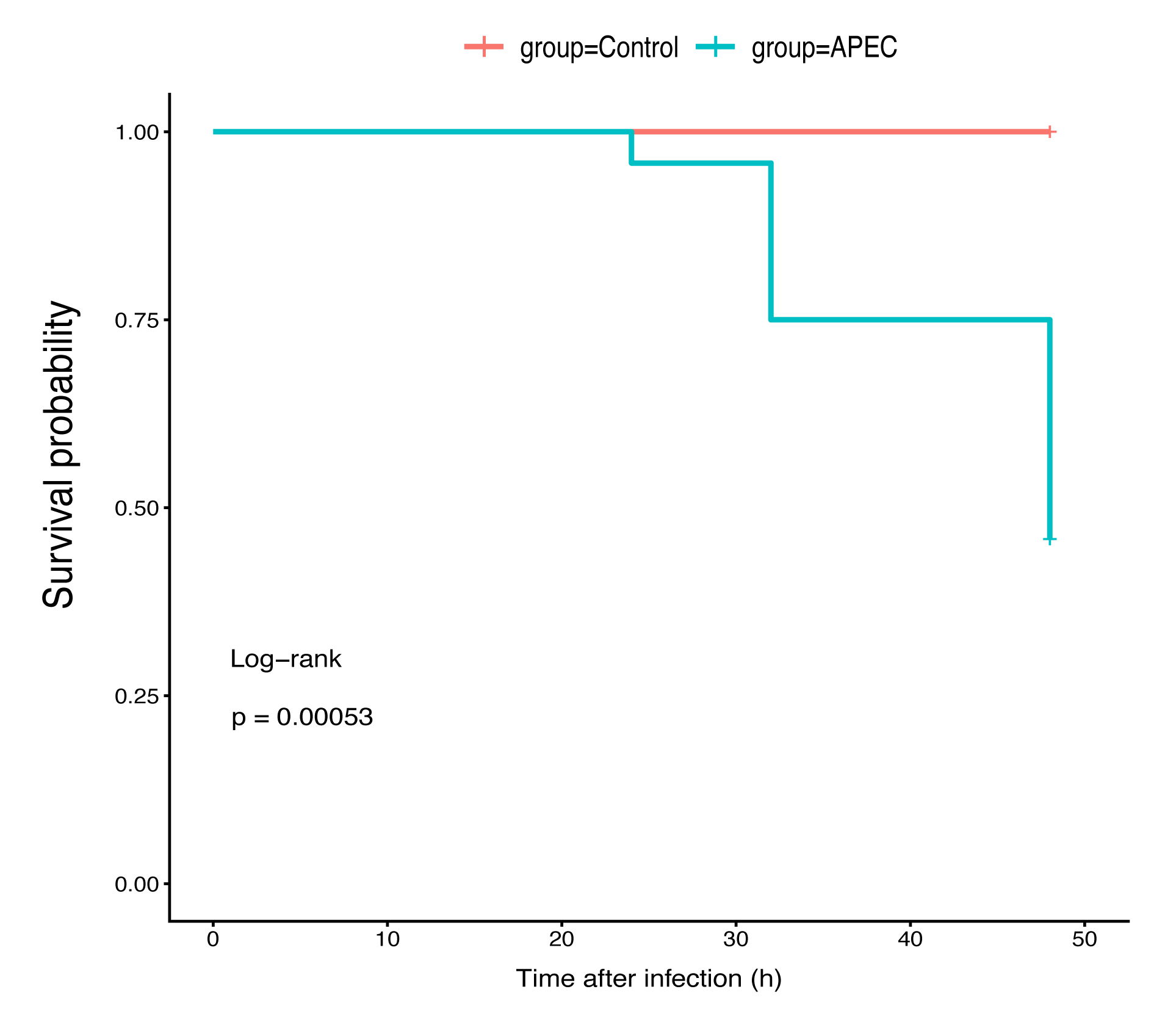

Supplement: Supplementary Figure S1 — Survival of chicken embryos following APEC infection. Embryo survival was monitored at 8-h intervals by candling from 0 to 48 h post-infection. Kaplan–Meier survival comparison was analyzed between the negative control and APEC-infected groups (Control, n = 16; APEC, n = 24). Curves were compared using the log-rank (Mantel–Cox) test (p = 0.00053). [file Image1.tif]

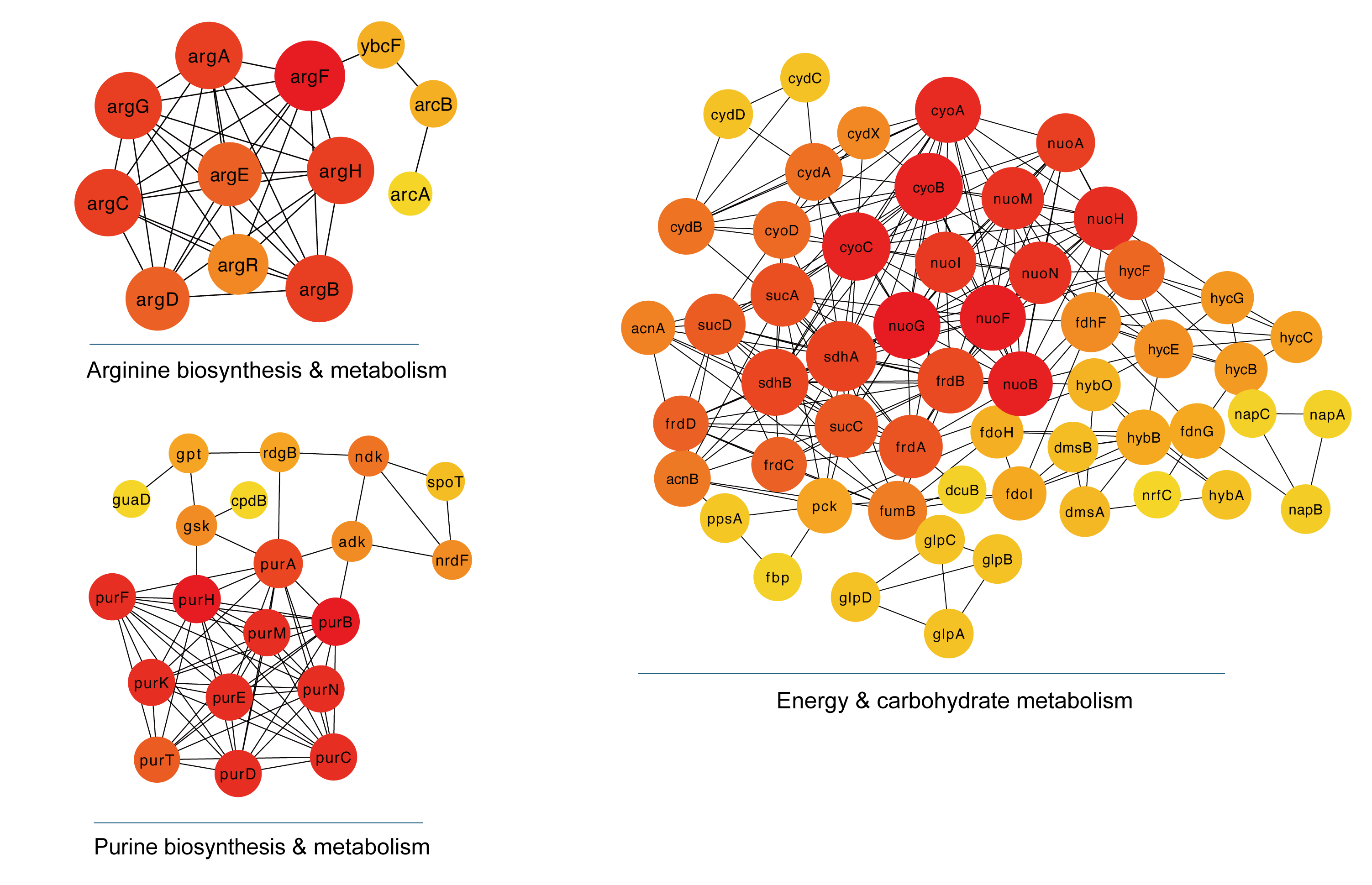

Supplement: Supplementary Figure S2 — Protein–protein interaction (PPI) networks of genes involved in selected metabolic pathways. The networks depict interactions among genes associated with arginine metabolism, purine metabolism, and energy and carbohydrate metabolism. Node size indicate degree centrality, with red representing highly connected proteins. [file Image2.tif]

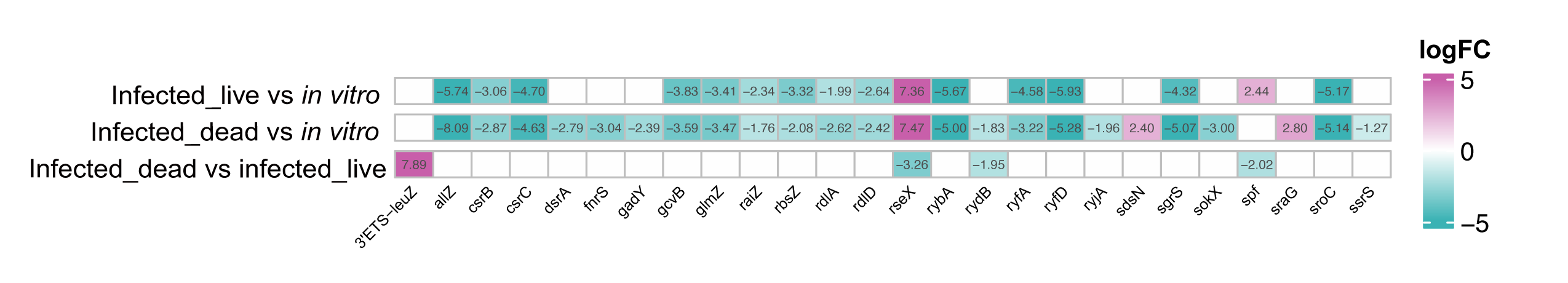

Supplement: Supplementary Figure S3 — Heatmap showing the differential expression of sRNAs in APEC from in vivo (infected-live and infected-dead) compared to in vitro culture. Log2 fold change (logFC) values that are statistically significant are shown. [file Image3.tif]
